# Authentication of milk thistle commercial products using UHPLC-QTOF-ESI + MS metabolomics and DNA metabarcoding

**DOI:** 10.1186/s12906-023-04091-9

**Published:** 2023-07-21

**Authors:** Ancuța Cristina Raclariu-Manolică, Quentin Mauvisseau, Renato Paranaiba, Hugo J. De Boer, Carmen Socaciu

**Affiliations:** 1 Stejarul Research Centre for Biological Sciences, National Institute of Research and Development for Biological Sciences, Alexandru cel Bun Street, 6, Piatra Neamț, 610004 Romania; 2 Natural History Museum, University of Oslo, P.O. Box 1172, Blindern, Oslo, 0318 Norway; 3 Natural Products Laboratory, School of Health Sciences, University of Brasília, Campus Universitário Darcy Ribeiro, Brasília, DF 70910-900, 70910-900 Brazil; 4 DNA Laboratory, National Institute of Criminalistics, Brazilian Federal Police, SAIS Quadra 7, Lote 23, Brasília, DF 70610-200 Brazil; 5 Faculty of Food Science and Technology, University of Agricultural Sciences and Veterinary Medicine, Mănăştur Street, nr. 3-5, Cluj Napoca, 400372 Romania; 6 BIODIATECH- Research Center for Applied Biotechnology in Diagnosis and Molecular Therapy, Trifoiului Street 12G, Cluj-Napoca, 400478 Romania

**Keywords:** Milk thistle, *Silybum marianum* (L.) Gaertn, Commercial products, Authentication, Quality, DNA metabarcoding, Metabolomics

## Abstract

**Background:**

Milk thistle is one of the most popular hepatoprotectants, and is often sold in combination with other ingredients. Botanical supplements are known to be vulnerable to contamination and adulteration, and emerging technologies show promise to improve their quality control.

**Methods:**

Untargeted and semi-targeted metabolomics based on UHPLC-QTOF-ESI^+^MS techniques, UV spectrometry, and DNA metabarcoding using Illumina MiSeq were used to authenticate eighteen milk thistle botanical formulations (teas, capsules, tablets, emulsion).

**Results:**

Untargeted metabolomics separated 217 molecules and by multivariate analysis the discrimination between the different preparations was established. The semi-targeted metabolomics focused on 63 phytochemicals, mainly silymarin flavonolignans and flavonoids, that may be considered as putative biomarkers of authenticity. All formulations contained molecules from silymarin complexes at different levels. The quantitative evaluation of silybins was done using in parallel UV spectrometry and UHPLC-QTOF-ESI^+^MS and their correlations were compared. DNA metabarcoding detected milk thistle in eleven out of sixteen retained preparations, whereas two others had incomplete evidence of milk thistle despite metabolomics validating specific metabolites, e.g., silymarin complex, identified and quantified in all samples. Meanwhile, the DNA metabarcoding provided insights into the total species composition allowing the interpretation of the results in a broad context.

**Conclusion:**

Our study emphasizes that combining spectroscopic, chromatographic, and genetic techniques bring complementary information to guarantee the quality of the botanical formulations.

**Supplementary Information:**

The online version contains supplementary material available at 10.1186/s12906-023-04091-9.

## Background

**Fig. 1:**
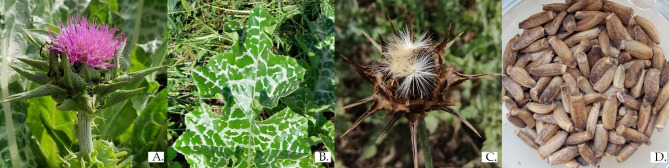
*Silybum marianum* (L.) Gaertn. **(A)** Red purple flower head with spiny bracts; **(B)** Variegated leaf with lobed margins; **(C)** Mature flower head with seeds; **(D)** Fruits. (Photos: A.C. Raclariu-Manolică; M. Naie)

*Silybum marianum* (L.) Gaertn. (Asteraceae, milk thistle, MT, Fig. 1) preparations are among the most commonly used botanical-based hepatoprotectants in complementary and alternative medicine [1–6]. Milk thistle has been purported to have also other health-promoting effects being used for the treatment of dyspeptic complaints, alcohol or drug-induced hepatic cirrhosis and fibrosis, and support treatment in hepatitis and other chronic inflammatory liver conditions [2, 7, 8], for stimulation of milk production in lactating mothers [9–11], and has been investigated for oncological indications and metabolic syndrome [2, 12, 13]. In Europe, intravenous silibinin, a flavonolignan isolated from milk thistle, has been approved as an antidote in patients intoxicated with *Amanita phalloides*, a mushroom that causes fatal poisoning [14].

The therapeutic activity of milk thistle is associated to a great extent with a mixture of flavonolignans, known as silymarin complex including mainly silibinin (or silybin) A and B, isosilibinin (or isosilybin) A and B, silychristin and silydianin [15–19]. These major flavonolignans together with the flavonoid taxifolin are considered the marker compounds for milk thistle identification [20]. Silymarin is present in seeds, fruits, and leaves of milk thistle, but mature seeds are being reported as having the maximum concentration [19, 21]. Milk thistle contains also other flavonoids (e.g. kaempferol, quercetin, rutin, luteolin, naringin, kaempferol, apigenin), proteins, sugars (arabinose, rhamnose, xylose, glucose), tocopherol, sterols (cholesterol, campesterol, stigmasterol, sitosterol), and lipids in the form of triglycerides (linoleic, oleic and palmitic acids) [22]. The putative mechanisms of action of the main bioactive compounds of milk thistle have been discussed by numerous pharmacological, pharmacokinetic, and toxicological studies [23–26].

Regarded to be “natural” and thus “safe”, thousands of botanical preparations are advertised, marketed, and sold via various channels, and are often preferred over synthetic pharmaceuticals by consumers [27, 28]. The claimed therapeutic properties, the high popularity among consumers, folk traditions, and the increased global market demand, have spurred studies on various aspects of botanicals and their derived preparations [29–31]. Ensuring their quality and safety and reducing the potential risks related to their intake are key priorities for these commodities, with a paramount focus on consumer health [32–36]. These matters have raised the interest in finding novel testing and quality monitoring strategies, including emerging technologies applied for authentication purposes [36, 37]. But, finding a single comprehensive analytical approach for the authentication of botanicals and their derived preparation is a complicated task hampered by the complexity of these products and by the lack of harmonization regarding regulations, definitions, and quality standards that vary between countries and continents [38–40]. Botanicals are inherent chemical mixtures prone to variability under natural conditions that are often reflected in the batch-to-batch composition variation of the final preparations [41, 42]. Moreover, they often have long and complex supply chains, where numerous ingredients are extracted and processed differently, and key aspects such as identity and authenticity remain challenging to assess, hindering accurate monitoring and quality control processes [42, 43]. These are only a few aspects that make botanical preparations some of the most vulnerable commodities worldwide, especially to accidental contamination and fraud through adulteration [44].

Treatments involving milk thistle are generally well tolerated in recommended doses, with a low incidence of adverse drug reactions and mild side effects when reported [23, 45, 46]. However, incorrectly used nomenclature for milk thistle-based material has generated a strong degree of skepticism regarding the safe use and efficacy of this botanical and its derived preparations [47, 48]. Regarding this, strong arguments have been made concerning the description of milk thistle compounds, particularly “silymarin” and “silibinin”, terms that were reported to be often used interchangeably [48]. Furthermore, the proportion of these marker compounds was reported as being prone to variability under natural conditions and significantly affected by the production and processing steps [47, 49–54]. Nevertheless, the chemical composition of the milk thistle used material was rarely determined in most of the studies focusing on its biological activity [52]. Thus, notwithstanding the purported beneficial role of milk thistle, the vague description of the chemical composition of the derived preparations represents probably, for several studies, the main pitfall in proving its clinical efficacy [52, 55, 56].

Moreover, widespread contamination with fungi, microbes, and pesticides has been reported in milk thistle-based dietary supplements raising serious safety issues for human health, as botanicals-induced hepatotoxicity may occur [55, 57, 58]. Studies have shown alarming discrepancies between declared and detected chemical content between brands of marketed milk thistle preparations, as well as within batches of the same preparations and manufacturers. These differences can critically alter the expected therapeutic effects [51, 55, 56, 59, 60]. Some of the studies focusing on the quantitative analysis of silymarin showed that a large number of investigated marketed preparations contained a lower amount than declared and some were even completely missing this marker compound of milk thistle [51, 53, 59, 61]. On top of this, some of the studies provided evidence of the presence of foreign matters in the products, reported as probably belonging to undeclared adulterants, but the identity of these adulterants was not determined [60, 62].

Considering the reviewed challenges, one may wonder if the consumption of commercial milk thistle preparations may rather be harmful than beneficial with regard to possible accidental contaminants and/or adulterants. Pharmacovigilance of these preparations remains difficult since they are sold over-the-counter with no medical prescription, and limited legislative framework to trace or monitor adverse reactions [63, 64]. In addition, the standard quality control analytical methods do not always have sufficient resolution for the identification of target plant species within complex preparations, and often are not able to detect non-targeted plant ingredients that may be present as contaminants or adulterants [39, 65]. Thus, new technologies and fit-for-purpose methodologies need to be adopted for the quality control of botanicals and their derived complex preparations [6, 36].

In this study, we propose and evaluate a novel analytical approach to investigate the authenticity of commercial botanical preparations labeled as containing *Silybum marianum* (L.) Gaertn. (milk thistle), either as unique ingredient or in combination with other plant-based ingredients. Using untargeted and semi-targeted metabolomics based on ultra-high-performance liquid chromatography coupled with quadrupole-time of flight mass spectrometry (UHPLC-QTOF-ESI^+^MS) data and ultraviolet spectroscopy (UV), alongside high-throughput DNA metabarcoding, we aimed to answer the following research questions: (1) Can UHPLC-QTOF-ESI^+^MS untargeted metabolomics unveil potential molecular markers that differentiate between unique and multiple ingredient milk thistle-based preparations?; (2) Can semi-targeted metabolomics identify key molecules to assess the authenticity of milk thistle preparations, and to detect any deviation compared to other plant ingredients stated on the label of the commercial preparation?; (3) Can UV spectrometry be useful as a fast method comparable to UHPLC-QTOF-ESI^+^MS for authentication of milk-thistle in botanical formulations?; (4) Can DNA metabarcoding be used to test for the presence of milk thistle in botanical preparations, and to detect the presence of off-label plant species? Ultimately, this study aims to provide a new fit-for-purpose complementary analytical approach to assess the quality of complex botanicals and derived formulations, to enable more rapid advances in the regulatory context.

## Methods

### Botanical formulations and reference material

Eighteen herbal preparations that included *Silybum marianum* (L.) Gaertn and or other derived compounds (i.e., silymarin) according to the label were randomly purchased from Romania (15) and Germany (3) in the autumn of 2021. The samples were bought from herbal shops (8), via e-commerce (6), retail stores (3), and pharmacies (1), and were sold as herbal teas (7), tablets (6), capsules (4), and one emulsion (Tables 1 and Additional file 1.A and 1.B). According to the label information, there were 7 unique ingredients (U) and 11 multi-plant ingredient preparations (M), as presented in Table 1. These products for scientific analysis were imported into Norway under Norwegian Medicines Agency license no. 18/13493–2. Each sample was given a specific ID number ranging from PA1 to PA18 and a code referring to the type and pharmaceutical form of the preparations, as follows: “U” unique ingredient, “M” multiple ingredients, “T” teas, “Tb” tablets, “C” capsules, and “E” emulsions. An overview of the samples including label information, but not the producer/importer name, lot number, expiration date, or any other information that could lead to the identification of that specific product can be found in Additional file 1.A. The five genuine MT herbal materials used as references for the identification and quantification of the main target compounds in the metabolomics analysis were kindly provided by the Agricultural Research and Development Station Secuieni (Neamt County, RO), Vegetable Research and Development Station Bacău (Bacău County, RO), and a local farmer from Fundu Tutovei (Bacău County, RO), and they have ID collection codes ranging from ACM24 to 27, and ACM29, and the code “Genuine U0” (their description can be found in Additional file 1.C.). Ancuța Cristina Raclariu-Manolică undertook the formal identification of the plant material used as a reference in this study. Voucher specimens are deposited at the National Institute of Research and Development for Biological Sciences,” Stejarul” Biological Research Centre (Romania), having deposition numbers ranging from PlantCheck_ACM24 to PlantCheck_ ACM27, and PlantCheck_ACM29, and available on request.

**Table 1:** Categories of herbal formulations (teas, capsules, tablets and emulsion) submitted to different analysis, and their codes applied for unique (U) or multiple (M) ingredients. Abbreviations: C-capsules; T-teas; Tb-Tablets; E-emulsion

Type of formulation	Unique ingredient (U)	Multiple ingredients (M)
**Teas**	PA7 (T2/U4)	PA1 (T1/M1)
	PA10 (T3/U6)	PA12 (T4/M6)
		PA13 (T5/M7)
		PA14 (T6/M8)
		PA18 (T7/M11)
**Tablets**	PA2 (Tb1/U1)	PA16 (Tb5/M10)
	PA3 (Tb2/U2)	
	PA5 (Tb3/U3)	
	PA9 (Tb4/U5)	
	PA17 (Tb6/U7)	
**Capsules**		PA4 (C1/M2)
		PA6 (C2/M3)
		PA8 (C3/M4)
		PA11 (C4/M5)
**Emulsion**		PA15 (E/M9)

### Extraction of phytochemicals

The same quantity of 5 g from each sample (5 genuine powdered MT and 18 herbal supplements) was suspended in 100 ml ethanol 70%, mixed 3 min by vortex and kept in an ultrasonic bath for 3 × 20 min at 50 °C. After the storage, 24 h at room temperature, each extract was centrifuged at 12,500 rpm and the supernatant was collected and filtered through 0.25 mm membrane filter. All extractions were made in triplicate.

### UV spectroscopy

The UV spectra (200–340 nm) were recorded using a UV/VIS Lambda 25 (Perkin Elmer Inc, Waltham, Massachusetts, USA) spectrometer and the measurements were done in quartz cuvettes, comparative to a blank sample (ethanol 70%). Each extract was filtered through a 0.4 μm nylon membrane and diluted with ethanol 70% in different proportions to fit in the spectral absorbance scale. The specific absorbances located in the region 286–288 nm were recorded to evaluate the levels of flavonolignans (FL). In parallel, a calibration curve was built with pure silybins A + B (25 to 75 micrograms/ml) having the following equation: y = 0.0246x–0.2363 (R^2^ = 0.9968), as presented in Additional file 2.A. Considering the calibration curve, the results for each formulation were expressed in mg silybin equivalents per g dry matter (d.m.). This method offered a preliminary information and a rough evaluation of the silymarin flavonolignans found in the herbal preparations that claimed their presence on the label.

### UHPLC-QTOF-ESI^+^MS metabolomics

#### Solvents, reagents, and analytical standards

HPLC grade pure ethanol, acetonitrile, and methanol were purchased from Merck (Darmstadt, Germany), and formic acid (99.99%) was purchased from Sigma-Aldrich (St. Louis, Missouri, United States). Deionized water was produced by a Milli-Q system (Millipore, Bedford, MA, USA). The analytical standard of Silybin (a mixture of silybin A and B) was purchased from Sigma Aldrich (CAS nr. 802918-57-6, St. Louis, Missouri, United States).

#### Untargeted and semi-targeted metabolomics

The metabolomic fingerprints of all ethanolic extracts were performed using the ultra-high-performance liquid chromatography coupled with electrospray -quadrupole-time of flight-mass spectrometry using the positive ionization (UHPLC-QTOF-ESI^+^MS) on a UltiMate 3000 UHPLC system equipped with a quaternary pump Dionex delivery system (Thermo Fisher Scientific Inc., Waltham, Massachusetts, USA), and mass spectrometry (MS) detection by a QqTOF MaXis Impact (Bruker Daltonics GmbH, Bremen, Germany). The metabolites were separated using a Kinetex column (Phenomenex Inc, Torrance, USA) (5 μm, 150 × 2.1 mm, 100 Å) at 25 °C. The flow rate was set at 0.8 ml·min^− 1^ and the volume of each injected extract was 8 µl. The mobile phase consisted of 0.1% formic acid in water (A) and 0.1% formic acid in acetonitrile (B). The gradient was: 20 to 40% B (0–5 min), 40–60% B (5–8 min), 60–70% B (8–10 min), 70–20% B (10–16 min), and 20% B isocratic until 24 min. Several quality control (QC) samples obtained from a pool of extracts were used in parallel to calibrate the separations. The chromatograms were processed using Chromeleon software (Dionex, Thermo Fisher Scientific Inc, Waltham, Massachusetts, USA). The MS parameters were: ionization ESI^+^, calibrated with sodium formate, capillary voltage 3500 V, nebulizing gas pressure of 2.8 bar, drying gas flow 12 l/min, drying temperature 300 °C. The control of the instrument and the data processing were done using the specific software TofControl 3.2, HyStar 3.2, Data Analysis 4.2 (Bruker Daltonics GmbH, Bremen, Germany).

#### Metabolomic data processing and statistical analysis

The Base Peak chromatograms and all MS spectra were recorded and processed by Compass DataAnalysis 4.2 (Bruker Daltonics, GmbH, Bremen, Germany) using the find molecular feature (FMF) algorithm. The time alignment, spectral background extraction, normalization by the median values, of the bucket values in analysis, and an 80% bucket filter were the used parameters. From the initial metabolic matrix, including retention time, MS peak intensity, signal/noise ratios (> 10), and mass-to-charge ratio (*m/z*) values of separated molecules, after retention of 60% common molecules, a total of 217 molecules, having *m/z* values from 270 to 615 Dalton, were selected.

The statistical analysis was done by the Metaboanalyst v5.0 *online* software [66] and algorithms (https://www.metaboanalyst.ca). From the matrices representing the MS peak intensity *versus* mass-to-charge ratio (*m/z*) values of each molecule from each sample the most relevant statistical parameters were tested to reflect the discrimination between sample groups, the prediction, and the correlation maps. Therefore, the Principal Component Analysis (PCA) and Sparse Partial Least Square Discriminant Analysis (sPLSDA), the Heatmaps and the Random Forest-based prediction were used to evaluate the similarities between samples, the identification of the putative biomarkers. According to the statistical analysis, molecules, which may explain the discriminations between samples and some specific putative biomarkers for authenticity, were selected and identified using international databases, e.g. Human Metabolome Database [67], Lipid Maps [68], Phenol-Explorer (version 3.6) [69], and PubChem [70].

As mentioned before, the untargeted analysis revealed 217 molecules to be considered for the discrimination between the different categories of formulations. In the second step, the semi-targeted analysis was performed using the same Metaboanalyst 5.0. software, One way ANOVA algorithm [66]. Sixty-three molecules were considered as potential authenticity markers, from phytochemical classes characteristic of MT seeds. Such semi-targeted analysis focused on silymarin complex, including taxifolin, but also were lignan precursors (coumaric acid and coniferyl derivatives), phytosterols, phenolic acids, flavonoids, fatty acids, and derivatives, as mentioned in Additional file 3.

For a quantitative evaluation of silybins in each formulation, using UHPLC-QTOF-ESI^+^MS technique, the calibration curve was also built using a stock solution of 4 mg/ml pure silybins A + B. Five different volumes of stock solution (from 1.25 to 7.5 µl were injected (corresponding to 5, 10, 15, 20, and 30 micrograms). The equation: y = 337240x + 108,540 (R^2^ = 0.9785) was considered to calculate the concentration of silybins in every sample, expressed in mg silybin equivalents per g dry matter curve (see Additional file 2.B).

### DNA metabarcoding

#### DNA Extraction

Each sample was already made into powder, and total DNA from all samples was extracted from the homogenized contents using the E.Z.N.A.®SP plant DNA kit (Omega Biotek Inc, Norcross Georgia) following the manufacturer’s instructions. DNA extracts were then quantified using a Qubit 2.0 Fluorometer with dsDNA Broad-Range assay kit (Invitrogen, USA). In the case of non-successful DNA extraction using the E.Z.N.A. Plant DNA Mini Kit, subsamples were extracted following a modified CTAB extraction method as described by Doyle and Doyle [71], and adapted by Raclariu et al. [72]. The final elution volume was 100 µl.

#### DNA libraries preparation and sequencing

All amplicon libraries were prepared in three technical replicates on 96-well polymerase chain reaction (PCR) plates. On each plate, we also included negative controls consisting of extraction blanks (created by performing all steps of the DNA extraction on “empty” samples) alongside the DNA extraction of other materials, and PCR controls (created by replacing the template DNA with ddH_2_O at the PCR step). This resulted in a total of 21 negative controls across the project (i.e., six extraction blanks analyzed in triplicate and four PCR controls). The amplicon libraries for the nuclear ribosomal target sequences, internal transcribed spacer nrITS2, were performed using indexed ITS-3p62plF1 and ITS-4unR1 primers designed in [73] following the indexing strategy as in [74].

PCR was conducted with the following conditions: 1X Q5 hot start high fidelity mastermix (New England Biolabs Inc, UK), 1X Q5 enhancer (New England Biolabs Inc, UK), 0.5 µM of each indexed ITS-3p62plF1 and ITS-4unR1 primer [73] and 3 µl of extracted DNA in a final volume of 25 µl. Unique dual-index primer combinations were used for each subsample as in [75], and the thermocycling protocol was as described in [73].

Amplicons were visualized on agarose gels and quantified using ImageLab Software v6.0 (Bio-Rad Laboratories, Inc., USA). Following quantification, uniform amounts of each amplicon were merged using a Biomek4000 liquid handling robot (Beckman Coulter, USA). The DNA library was then cleaned using 1.0X AMpure beads (Beckman Coulter, USA), size selected using BluePippin (Sage Science, USA), and quantified on a Fragment Analyzer (Advanced Analytical Technologies, Inc., USA) using the High Sensitivity Genomic DNA Kit (Agilent). The library was finally sequenced on MiSeq platform v3 (Illumina, San Diego, CA, USA), alongside samples from other projects.

#### Bioinformatics data analysis

Bioinformatic processes for the metabarcoding analysis were conducted as in the annotated scripts provided on the following GitHub page: https://otagomohio.github.io/workshops/eDNA_Metabarcoding. In brief, forward and reverse raw sequencing files obtained following MiSeq sequencing were merged using PEAR 0.9.3 [76] and demultiplexed using the ngsfilter command from the OBITools software suite [77]. The obigrep command was used to select fragments > 400 bp, to check, and further quality filtering was conducted to remove sequences < 100 bp using the fastq_filter command from the USEARCH algorithm [78]. Sequences were then dereplicated using the fastx_uniques command from the USEARCH algorithm [78], and sequences with less than 10 occurrences in the dataset were removed. Our dataset was then denoised using UNOISE algorithm (i.e. unoise3 command from USEARCH) [79], and ZOTUs (e.g., Zero-radius Operational Taxonomic Units) were retrieved. Finally, the taxonomic assignment was performed using the blastn command from the BLAST + application [80]. We applied strict filtering control to remove any false positive detection. For each sample, we first selected all ZOTUs corresponding to a unique species and discarded the ZOTUs that didn’t have at least two reads in at least two PCR replicates. Then, for each retained ZOTUs, we subtracted the highest number of reads which could be found in the corresponding ZOTU in any of all negative controls (extraction blanks and PCR controls). This conservative approach was applied in all PCR replicates of the sample. This was manually done for each analyzed sample of our study. We chose this approach to ensure that potential contamination or “tag-jump” will not lead to potential false positive results. Finally, ZOTUs were manually checked and all ZOTUS corresponding to a unique species were pooled together in a unique species identifier, and the read numbers were added (see Additional file 4.A. and B.) to avoid overinflation of the species diversity detected in this study.

## Results

### Metabolomics

#### Phytochemical fingerprinting by untargeted UHPLC-QTOF-ESI^+^MS metabolomics

**Fig. 2:**
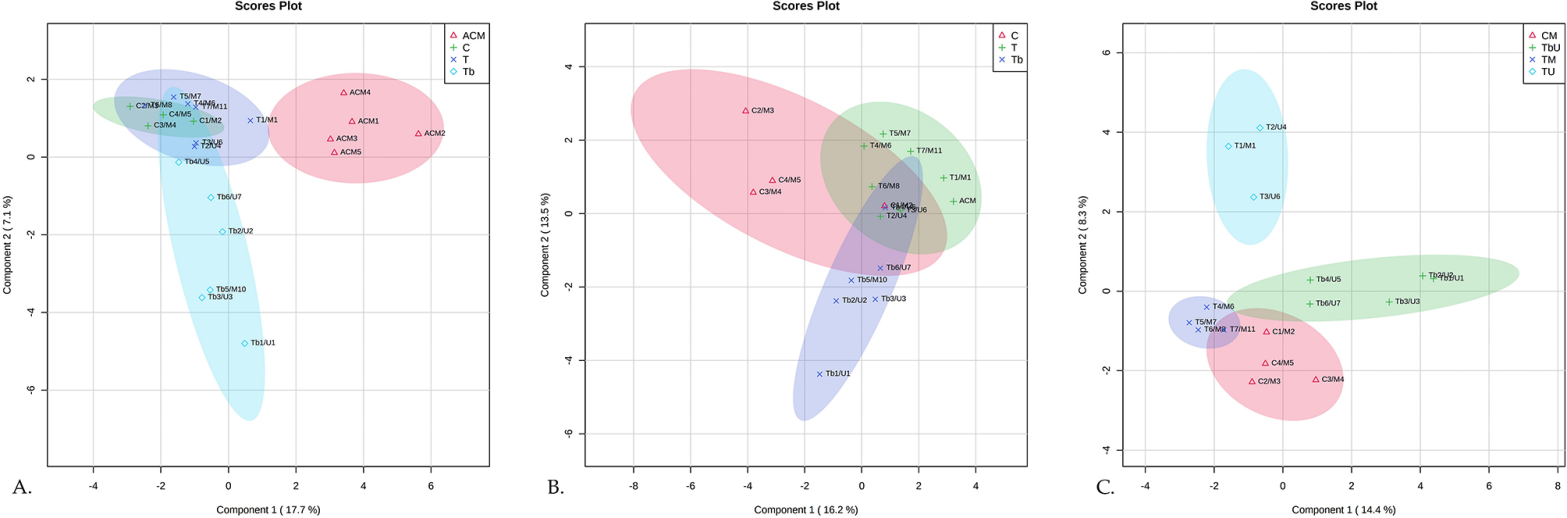
The sparse PLSDA score plots showing the discrimination between the different categories of herbal preparations. **(A)** Comparison of the fingerprints specific to genuine MT seeds (ACM), teas (T), capsules (C), and tablets (Tb) claiming to contain MT ingredients, as unique or multiple combinations. **(B)** Discrimination between the fingerprints of capsules (C), teas (T), and tablets (Tb). **(C)** Discrimination between the fingerprints of the herbal preparation containing unique (U) and multiple (M) ingredients

Multivariate analysis was first applied to discriminate between the five MT genuine samples (code ACM) *versus* herbal formulations commercialized as teas (T), tablets (Tb), or capsules (C) containing MT as a unique ingredient (U), or in combination with other plant-based ingredients (M), in agreement with the information stated on their labels, as presented in Table 1 and Additional file 1.A. A total number of 217 molecules were separated and included in the matrix for multivariate analysis using Metaboanalyst v5.0 software. Figure 2.A., 3B., and 3 C. show the sPLSDA score plots for the different categories of herbal formulations. First, a comparison between the fingerprints specific to genuine MT seeds (ACM), teas (T), capsules (C), and tablets (Tb) claiming to contain MT ingredients are presented in Fig. 2.A. A significant discrimination was observed between the genuine MT seeds and formulations, with a co-variance of 24.8% for the first 2 components. The Tb group was significantly different in this case, also showing a higher heterogeneity among the six formulations. The C and T groups were partly superposed since capsules and teas contain powders of raw plant tissues. These two groups were discriminated against the genuine ACM group (MT seeds) since they contained other added ingredients and excipients.

Further, it was seen the discrimination between the fingerprints of capsules, teas, and tablets, therefore similar score plots were obtained, with a co-variance of 29.7% (Fig. 2.B.). Here, the difference between the composition of tablets vs. teas or capsules was more visible. According to Additional file 1.A., one can see that tablets, more than capsules, include non-herbal ingredients (standardized extracts, dextrins, flavonoid pigments) which may explain this discrimination. The influence of unique (U) versus multiple (M) ingredients upon the discrimination between capsules, teas, and tablets was also plotted, the co-variance being 22.7% for the first two components (Fig. 2.C.). Here, also a clear discrimination between TU and TM subgroups of teas, containing unique and multiple ingredients was noticed. This shows that the addition of other plant phytochemicals in tea can be easily identified by untargeted metabolomics. Additionally, the capsules and teas with multiple components have similar fingerprints, different from tablets.

### Semi-targeted metabolite profiles

The semi-targeted analysis focused on specific groups of molecules that were previously identified in MT seeds and formulations, namely flavonolignans including silymarin complex, taxifolin, lignan precursors (coumaric acid and coniferyl derivatives), phytosterols, flavonoids, phenolic acids, fatty acids and polar lipid derivatives, a number of 63 molecules being selected and identified (see Additional file 3) using the match of m/z values with HMDB and other databases ( as mentioned in Materials and Methods).

**Fig. 3:**
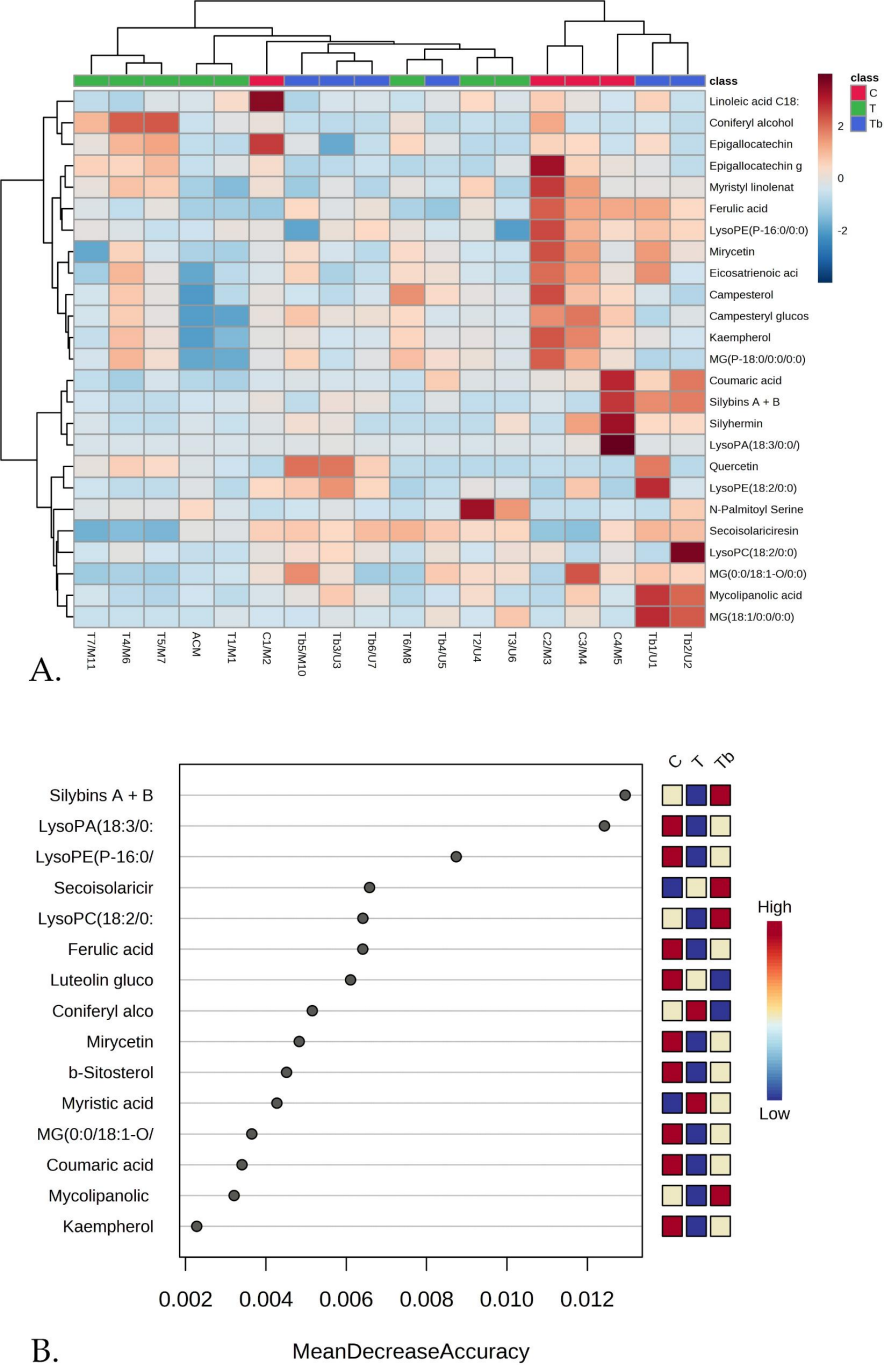
**(A)** The Heatmap showing the clusters of the three groups of samples C, T, and Tb vs. the top of 25 molecules selected as most relevant for the discrimination between the teas (T/U vs. T/M), four multiple ingredient capsules (C1-C4/M2-M5), and tablets T/U, T/M. **(B)** The RF analysis plot showing the top of 15 molecules to be considered as potential biomarkers, according to the Mean Decrease Accuracy value

The multivariate analysis focused on the most common molecules that may discriminate and characterize the profile of individual teas, capsules, or tablets. Figure 3.A. presents the heatmap of sample clusters (T and ACM-green, C-red, Tb-Blue) vs. the main 25 molecules responsible for the discrimination, as selected by Metaboanalyst algorithm. Specific MT molecules like silybins A + B and silyhermin are readily identified, as well as phenolic acids, flavonoids, and polar lipids as putative biomarkers for discrimination. Some capsules (C1/M2, C2-C4/M3-M5) and tablets (Tb1/U1 and Tb2/U2) showed specifically higher levels of such molecules. The Random Forest (RF) analysis (Fig. 3.B.) classified the top 15 molecules to be considered most significant as putative biomarkers, according to Mean Decrease Accuracy (MDA) values > 0.002.

Considering the mean values per formulation, one can see that, for example in the Tb group, Silybins A + B had significantly higher levels, followed by capsules (C) and teas (T). Considering all 15 molecules, the highest levels of phytochemicals were found in capsules (C) with 9 out of 15 molecules. Focusing on these 15 to 25 molecules, out of the 63 separated and identified by UHPLC-QTOF-MS, would be an effective approach for developing qualitative or quantitative evaluation of these herbal supplements.

### Profiles of silymarin complex

**Fig. 4:**
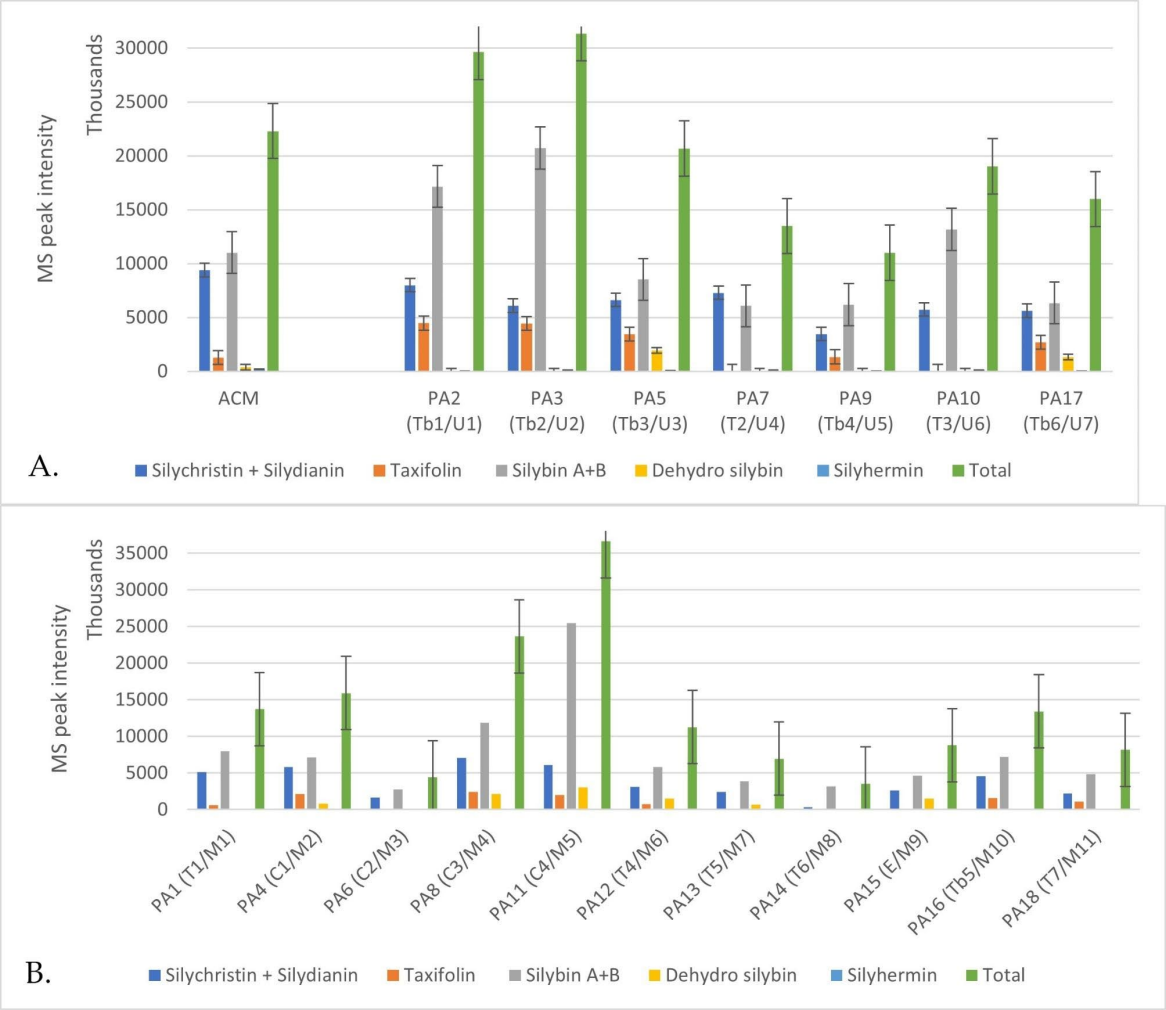
**(A)** Comparative values of the mean MS peak intensities recorded for the genuine MT seeds (ACM) comparative to herbal preparations (teas – T; tablets- Tb), which declared to have a unique ingredient (U). **(B)** Comparative values of MS peak intensities for the herbal preparations (teas – T; tablets- Tb; capsules-C; emulsion-E) with multiple ingredients (M). The error bars (± SD) from triplicate measurements represented 20–30% of the mean values represented in the graphic

Since silymarin flavonolignans are specifically related to genuine MT products, a more targeted analysis focused on this subclass of molecules and compared their levels in the individual products with unique (U) or multiple ingredients (M). Figure 4.A. and 4.B. show the MS peak intensities of the silymarin class of molecules, which may be considered as relevant biomarkers of product quality and authenticity. The targeted silymarin flavonolignans were silybins A + B, silychristin and silydianin, taxifolin, dehydrosilybin, silyhermin. The total intensity, as the sum of all silymarin flavolignans was also calculated, as presented in green columns.

Silybins were identified in all samples followed by silychristin and silydianin. In the group U (unique ingredient) of samples (Fig. 4.A.), the richest preparations were Tb2/U2, Tb1/U1, Tb3/U3, Tb6/U7 followed by T3/U6, and T2/U4. The sample Tb4/U5 had the smallest content, but still with an acceptable content of silybins, almost 50% of the genuine samples (the mean value from the ACM group was considered). Taxifolin, silychristin and silydianin were found in higher levels especially in samples Tb2/U2, Tb1/U1, and Tb3/U3.

In the group M (multiple ingredients) of samples (Fig. 4.B.), the richest preparations were C4/M5, C3/M4, and C1/M2 followed by T1/M1 and T4/M6, Tb5/M10, E/M9, T7/M11. The samples C2/M3 and T6/M8 had the smallest content of silybins. Silychristin and silydianin were found in C3/M4, C4/M5, C1/M2, and T1/M1, at levels representing around 60% of the levels found in the samples with unique ingredients. Taxifolin was also found, but at lower levels.

Generally, tea products with multiple ingredients contained lower levels of silymarin complex. Meanwhile, the capsules and especially tablets contained higher levels of silymarin complex probably due to the use of standardized, concentrated MT extracts as ingredients, as can be seen in samples Tb1-Tb3, C3, and C4. Taxifolin was identified at higher levels in tablets Tb3-Tb6, but also in some teas and capsules.

As expected, the ratio between the silymarin complex in the U vs. M group was around 2, as presented in Additional file 5. A significant variability was noticed, explained by the different ingredients used and claimed on the label and the type of formulation (teas vs. tablets vs. capsules).

To authenticate by accurate analysis is still difficult since, as can be seen in Additional file 1.A., the producers of these herbal formulations did not report the concentration of active compounds belonging to the silymarin complex on the product labels. Although semi-targeted analysis could confirm the presence of silymarins as well as their relative levels, an accurate comparison with the claimed composition mentioned on the label was not possible.

### Quantitative evaluation of silybins using UV-spectrometry and UHPLC-QTOF-ESI^+^MS

The semi-targeted analyses showed that the silymarin complex compounds were especially suitable as quality indicators. In order to further evaluate these compounds for authenticity assessment, calibrations curves were built with analytical pure standards of silybins A + B, using UHPLC-QTOF-ESI^+^MS for accurate calculation of silybins and UV spectrometry (as a fast, less accurate but indicative method of silymarin complex-absorbing molecules in ethanol at 288 nm) as presented in Additional file 2.A.

**Fig. 5:**
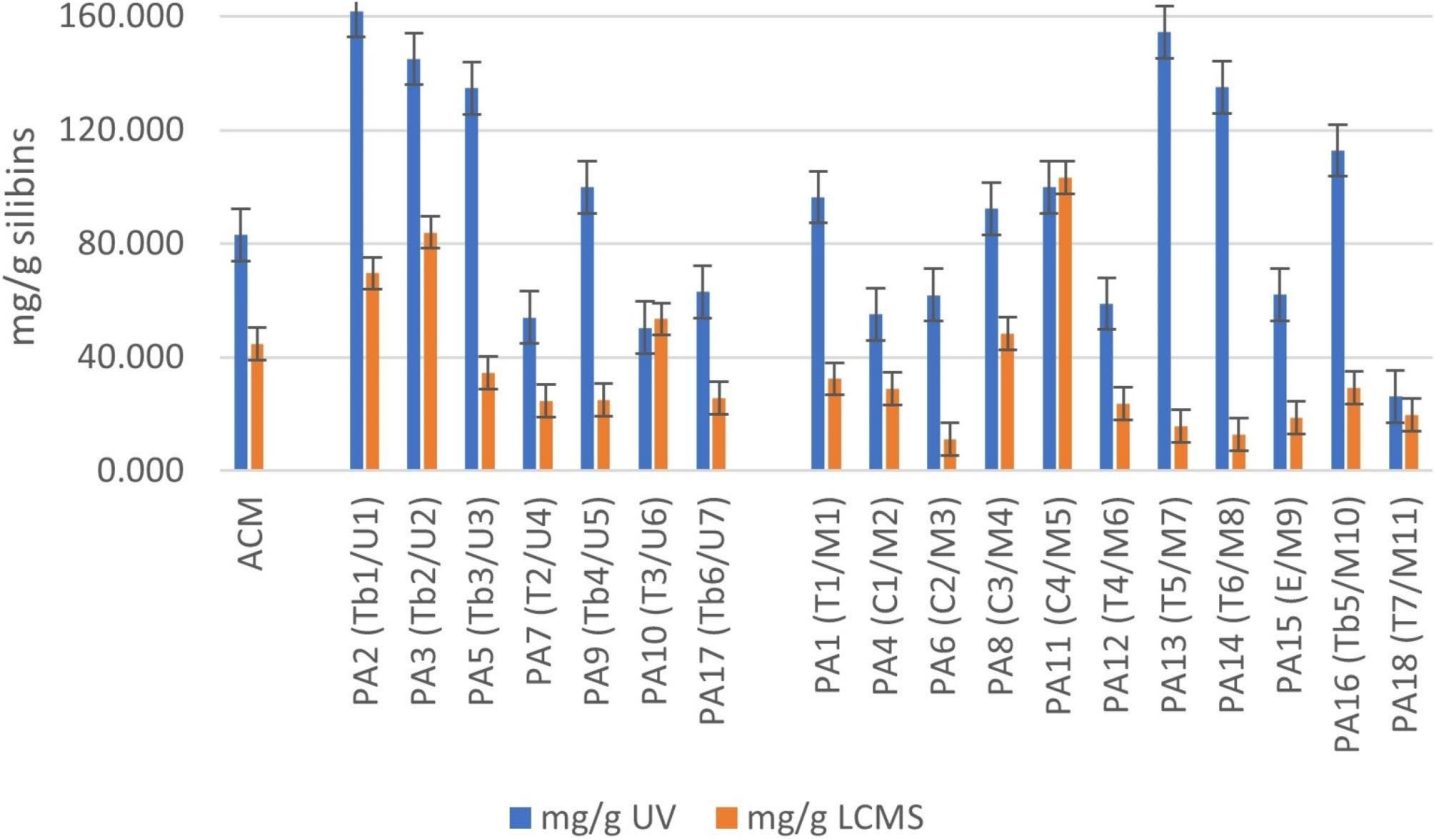
Comparative evaluation of silybins concentrations (mg silybins/g sample) in all herbal preparations, as determined by UV spectrometry and UHPLC-QTOF-ESI^+^MS analysis. For codes and abbreviations see Table 1 and Additional file 1.A. The error bars (± SD) from triplicate measurements represented 20–30% of the mean values represented in graphic

Based on the calibration curves, a comparative evaluation of silybins concentrations (mg/g d.m.) in all herbal preparations, as determined both, by UV spectrometry and UHPLC-QTOF-ESI^+^MS analysis is presented in Fig. 5.

With only a few exceptions (samples T3/U6 and C4/M5), the silybins concentrations determined by UHPLC-QTOF-ESI^+^MS were around 2–3 times lower compared to data released by UV spectrometry in unique ingredient samples (group U) and up to 10 times lower in multiple ingredient samples (group M), respectively. This is explained by the UV absorbance of other phenolic derivatives, besides silybins, at 288 nm. Therefore, the UV analysis overestimates the silybins concentration and is indicating mostly the pool of flavonoids, including flavonolignans. Meanwhile the UHPLC-QTOF-ESI^+^MS, gives a much more accurate information, regarding the types and levels of the different molecules, offering a more complete picture of the identity of the products. Nevertheless, these results show that UV-spectrometry can be applied as a preliminary, rough evaluation of the formulation, whereas UHPLC-QTOF-ESI^+^MS may target more precisely the molecules to be authenticated and also other components, in a semi-targeted or quantitative way.

However, in this study, the information provided on the label by the producers was not precisely mentioned, and in many cases lacking important details. For instance, it was not always clearly defined what the proportions were between an ingredient such as MT standardized extracts and additional powdered MT seeds, or the concentration of key-compounds responsible for the claimed effect, e.g., other ingredients added in the preparation.

### DNA metabarcoding

Qubit fluorometer quantitation showed large differences in total DNA concentrations among the eighteen analyzed MT-based botanical preparations (see Additional file 6). The sequencing success rate was 100% (18/18 samples). A dataset consisting of 1,163,356 reads fulfilling our initial trimming and filtering quality criteria was obtained, with an average of 77,557 reads per sample. Zero-radius operational taxonomic units (ZOTUs) were obtained for all preparations (100%) (see Additional file 4.A.). Sixteen preparations (89%) had ZOTUs that passed bioinformatics trimming and filtering quality criteria that require ZOTUs to have at least 10 reads in the whole dataset and at the samples level, more than one read and being detected in at least 2 out of 3 replicates in order to be retained for further analysis. Two samples (tablets PA2 and PA5) did not fulfill the imposed criteria and were excluded (i.e., there was no read remaining for any ZOTU following the various filtering steps). ZOTUs and their read numbers for the same species were merged for further analysis. Across all sixteen retained samples, a total of 59 different species (declared and non-declared on the label), were identified using the basic local alignment search tool (BLAST) from the retained ZOTUs.

The main targeted plant ingredient - *S. marianum* (milk thistle), was detected in eleven out of sixteen retained preparations. Out of the five single ingredient samples - those containing only *S. marianum* according to the label, *S. marianum* was detected in four samples *(*PA3, PA7, PA9, PA10) and in one not (PA17). Out of eleven multiple ingredient samples - those containing *S. marianum* together with other species according to the label, *S. marianum* was detected in seven samples (PA1, PA6, PA8, PA11, PA12, PA14, PA15) and in four not (PA4, PA13, PA16, PA18). The fidelity for *S. marianum* in single-ingredient products was 80% (4 out of 5), and for multi-ingredient products, 64% (7 out of 11) (see Additional file 7.A and 7.B).

All five retained single-ingredient samples contained species not mentioned on the label. Two of the multi-ingredient samples contained all species listed on the label (the capsules PA6 and PA8), but both also contained off-label species, and seven contained fewer species than listed on the label (PA1, PA4, PA11, PA12, PA14, PA15) and apart from PA14 they all contained additional off label species. Two samples contained none of the species from the label (PA13, PA16, PA18) but instead contained off-label species. The overall ingredient fidelity (detected species from product label/total number of species on the label) for multi-ingredient products was 45% and for all products 56% (see Additional file 8).

**Fig. 6:**
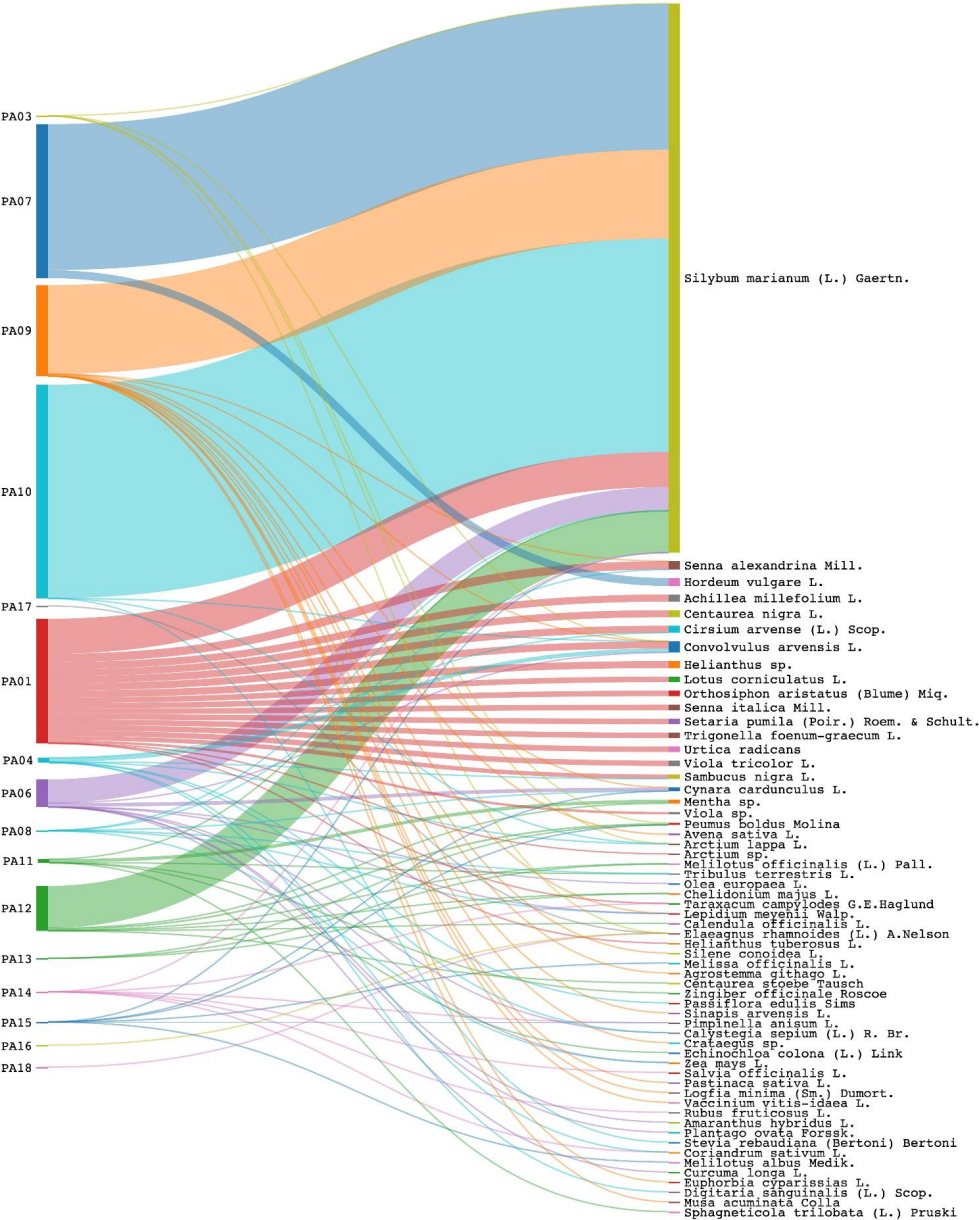
Sankey diagram summarizing detected species (declared and non-declared on the label), from the retained samples, using DNA metabarcoding. Only species represented by ZOTUs detected in ≥2 replicates and with ≥2 reads are shown. Sizes of flows denote proportions of reads at the species level

A total of 47 species non-listed on the label were detected. The most abundant species by sequence reads were *Hordeum vulgare* L., *Urtica radicans* Wight, Viola sp. (*Viola arcuata* Blume; *Viola arvensis* Murray), *Avena sativa* L., and Helianthus sp. (*Helianthus divaricatus* L.; *H. giganteus* L.; *Helianthus grosseserratus* M.Martens). The plant taxa detected in the samples are presented in Fig. 6.

### Comparative results

The results from the UHPLC-QTOF-ESI^+^MS untargeted analysis gave initial valuable information about the general fingerprint of the different categories of formulations, identifying a large number of molecules (217) that can be used as specific indicators of genuine ingredients (MT seeds and other plant components) and subgroups of herbal formulations, such as tablets that may have different fingerprints due to the more complex non-herbal ingredients. Nevertheless, the untargeted analysis does not offer enough information to make a more precise authentication of individual products and to find the relevant biomarkers for their identity.

The semi-targeted analysis, which focused on 63 molecules belonging to relevant phytochemicals for MT identification, gave better indications for the key molecules to be considered as authenticity biomarkers. The silymarin complex compounds (silybins and taxifolin) showed to be relevant as biomarkers of authenticity. The quantitative evaluation applied comparatively, using UV-spectrometry (based on absorbance at 288 nm and expressed in silybin-equivalents) and the accurate determination of silybins by UHPLC-QTOF-ESI^+^MS showed different contributions of MT-based ingredients in unique-ingredient products and multiple-ingredient products.

DNA metabarcoding had a good resolution in detecting MT at the species level and provided insights into the total species composition of herbal preparations labeled as containing unique (U) and multi-ingredients (M). The DNA metabarcoding and LC-MS semi-targeted metabolomics results were in accordance for eleven samples (61%). LC-MS could validate the presence of S. marianum in another five samples (100%) that have not passed the filtering criteria of DNA metabarcoding analysis.

## Discussion

It is well-accepted that botanicals and their derived herbal supplements are susceptible to various issues that raise serious quality and safety concerns [33]. Challenges may occur throughout the value chain, from cultivation or wild harvesting of the medicinal plants as sources of raw material to the final marketed product [35, 81].

In spite of the less exigent legislative regulations regarding herbal supplements, many laboratories apply new analytical approaches for the analysis of milk thistle content in raw materials and/or derived herbal formulations, including high-performance liquid chromatography (HPLC) with different types of detectors, thin layer chromatography (TLC), high-performance thin layer chromatography (HPTLC), hyphenated mass spectrometry, but also UV spectrometry [17, 52, 55, 82, 83]. However, milk thistle preparations are often highly processed and usually mixed with other plant ingredients, limiting the accuracy of traditional analytical methods in identifying the targeted plant species, and making it even more challenging to detect non-target species. Hence, applying new fit-for-purpose technologies and methodologies will perhaps enable a more accurate quality assessment of milk thistle-derived preparations [36, 65]. In this study, we combined two emerging technologies - metabolomics and DNA metabarcoding for the authentication of milk thistle-based preparations.

Generally, metabolomics is defined as the holistic qualitative and quantitative measurement of the complete set of small metabolites in a biological system at a given time [84–86]. As one of the most rapidly evolving fields, metabolomics found its applications in a plethora of basic and applied studies of the life sciences [87, 88]. Recent innovation and progress in metabolomics technologies have been used also to address a wide range of biological questions within the field of natural products [89–92]. These developments have opened new perspectives in the field of botanicals and derived herbal preparations by providing, among others, powerful tools for their authentication and quality assessment [37, 93]. Metabolomics comprises methods and advanced analytical platforms, with a high degree of sensitivity, selectivity, and reproducibility that enable a broader insight into the highly diverse metabolome complexity [94, 95], as already shown in several studies focusing on metabolic profiling in complex mixtures [96, 97]. Nevertheless, each analytical method has its own advantages and disadvantages, and the choice of a certain method is typically driven by the focus of the study, followed inter alia by the nature of the samples, costs, or accessibility [96–99]. UHPLC-QTOF-MS using ESI^+^ fragmentation is a versatile technique that imparts great promise for the comprehensive authentication of botanicals and botanical preparations and was the method of choice in this study. Here, untargeted and semi-targeted metabolomics as well as a quantitative evaluation of silybins as biomarkers of MT presence in different herbal formulations have been used and compared for their analytical efficacy in the context of authenticity and quality control of milk thistle-derived commercial preparations.

While targeted metabolomics has limited coverage of the metabolome as it aims to measure a predefined set of known metabolites, untargeted metabolomics focuses on a rather wider coverage, or ideally, complete measurement, of the relative levels of metabolites in a sample [100], enabling the simultaneous comparison of several samples without having *a priori* information about their content or suspicion of contamination or adulteration [84]. Here, by untargeted approach, there were identified molecules that can compare unique (including genuine samples) ingredient samples against multi-ingredient MT preparations and various subgroups of herbal formulations, in an attempt to find possible metabolites to be used as key markers in the authentication process. However, with the untargeted approach, it was not possible to find a single precise authentication of the products. Meanwhile, semi-targeted metabolomics is a promising, alternative, enabling the measurement of predefined metabolites known to reflect the presence of MT [101] e.g. flavonolignans. In our study, the semi-targeted analysis focused on 63 relevant phytochemicals and gave good indications regarding the key molecules to be further considered as authenticity markers, the silymarin complex (silybins and taxifolin) being the most relevant. MT was found to be present in all preparations, in agreement with the genuine MT seed composition, but at different levels. The comparative evaluation using both UV spectrometry and UHPLC-QTOF-ESI^+^MS showed different contributions of MT-based ingredients. These findings corroborate previous results showing various degrees of substitution and adulteration of the botanicals [102–108].

However, the usefulness of metabolomics based on specific phytochemicals is still a challenge for herbal product authentication and has a limited capacity to detect other botanical ingredients e.g. contaminants or adulterants [37].

While metabarcoding is focused on ingredient authentication by DNA recognition (qualitative), the metabolomic approach is looking at molecules that can be found either in the key ingredient (to check its presence and quantity in the product) or in other ingredients (which are usually found in plant mixtures of teas, powders of standardized extracts in capsules or tablets, etc.) or even non-declared excipients. Here we tried to see the capability of the metabolomic approach to identify similarities and differences between these products compared to genuine plant metabolites. Also, we followed in parallel the classical “phytochemical analysis” identifying and quantifying just the molecules belonging to the silymarin complex, e.g., silybins A + B and taxifolin.

DNA metabarcoding brings together the innovation of high-throughput sequencing (HTS) technologies and the DNA barcoding concept, enabling simultaneous multi-taxa identification from a pool of genetic material containing DNA from different origins [109, 110]. This approach generated an emerging area of research with practical applicability in the analysis of species composition of a wide range of multi-ingredient and highly processed samples, being used today in regulatory, conservation, and commercial contexts [38, 111–115]. This has lately emerged as a cost-effective and reliable method to improve the authentication and quality control process of botanicals and derived preparations [38, 65, 116].

Here, DNA metabarcoding results indicate a high level of inconsistencies between the identified species and those listed on the labels of the sixteen retained preparations. The targeted plant ingredient, milk thistle, was detected in 11 (68,75%) out of sixteen retained preparations, including three unique and eight multi-ingredient preparations (five herbal teas, two tablets, three capsules, and one emulsion). However, we emphasize that in four other preparations (PA2, PA4, PA5, and PA13) MT was detected – but we couldn’t validate its positive identification since the samples did not fulfill the imposed bioinformatics quality requirements. Either way, semi-targeted metabolomics confirmed the presence of MT in all four products. Six preparations contained all the listed plant-based ingredients (P3, P6, P7, P8, P9, P10), but additional plant species were detected in all of them. According to the label, this included four unique and two multiple ingredients (two tablets, two capsules and two herbal teas) preparations. Two samples (PA2 and PA5) did not fulfill the trimming and filtering quality criteria and they were not considered in the results and discussion. The findings corroborate previous studies, showing significant incongruences between the detected species and those listed on the labels of some marketed botanical preparations [72, 117–122].

Considering the nature of sourced botanicals and the long value chain to the final preparation, the discrepancies between the species detected using DNA metabarcoding and those listed on the product labels require a careful evaluation regarding the possible sources of contamination or adulteration. In this study, we used the information found on the label/leaflet for each preparation to define some variables that can impact the interpretation of the results. Thus, the evaluation of the authentication results was made in line with *a priori* information such as the origin and cultivation conditions, and *a posteriori* information such as the taxonomic identification of ZOTUs. In this regard, we highlight also that DNA metabarcoding is a very sensitive method, and even traces of another species or a pollen grain will give a positive identification. For instance, in this study, various wind-pollinated plant species (anemophilous) were detected and their presence in our results can be expected and considered as normal trace contamination – if they are in quantities that do not pose any quality issues of the preparation and if they are within the permitted contamination range. However, quantifying the relative species abundance within a sample is beyond the technical characteristics of DNA metabarcoding since many potentially confounding factors can affect read numbers, thereby, in these cases, appropriate phytochemistry methods should be used on a punctual-based evaluation when contamination or adulteration is suspected. The off-label detected species can be interpreted as contamination or adulteration, but also as being generated by amplification bias (i.e. PCR chimeras), sequencing errors, or false-positive taxonomic identifications due to errors in the barcode sequences reference databases [123–125]. To mitigate false positives and to increase the overall reliability of results, in this study we used technical replicates based on three independent PCR amplified products from the same preparation. Further, very strict filtering and trimming thresholds for sequence reads were applied to overcome sequencing errors, followed by very conservative selection standards in order to validate a positive identification. The varying degrees of success in identifying the species listed on the labels can be due to a failure to detect them with metabarcoding. This can be explained by false negatives that are often due to a combination of highly degraded DNA resulting from harvesting, drying, storage, transportation, and processing [119, 126, 127], the inability of recovering DNA due to the presence of pharmaceutical excipients affecting DNA extraction [128], or as result of poor primer fit and amplification biases [129], stochasticity due to low DNA concentrations [130], or incomplete reference databases.

Proper analytical validation of DNA metabarcoding is necessary before this can be implemented for molecular diagnostics, both in quality monitoring programs in a regulatory context, and in supply chain management systems by the industry sector. Important steps have been taken toward validating and standardizing DNA metabarcoding for quality control in commercial applications and regulatory contexts. A very good practical example is the study commissioned by the Federal Office of Consumer Protection and Food Safety (BVL) in Germany [131]. In this study within an inter-laboratory ring trial including 15 laboratories, the reproducibility, robustness, and measurement of DNA metabarcoding uncertainties, have been analyzed using meat-based multi-ingredient samples. The study concluded that DNA metabarcoding is a robust authentication tool and can be used in routine analysis by official food control laboratories [131]. While some DNA barcoding methods are validated and standardized for quality control in commercial applications and regulatory contexts [132], so far, no similar large inter-laboratory DNA metabarcoding protocols were performed for its validation as an authentication tool in the field of botanicals and their derived preparations. Even if DNA metabarcoding addresses a number of limitations when using plant-based samples, we expect that a common effort for a validation study will be performed and propose a DNA metabarcoding protocol applicable to the quality control systems of botanical preparations.

The application of emerging and innovative techniques and fit-for-purpose methodologies to advance the evaluation of botanical preparations in the context of quality assessment is strongly advocated today [36, 133]. Each analytical technique has its benefits and limitations, and interdisciplinary approaches have been shown to improve the quality assessment process of botanical preparations [65, 116, 133, 134]. The results of this study corroborate previous results confirming the advantages of combining analytical approaches for the quality assessment of botanical preparations [72, 121, 135].

## Conclusions

This study used untargeted and semi-targeted metabolomics analysis based on UHPLC-QTOF-ESI^+^MS data and UV spectrometry, alongside high-throughput DNA metabarcoding using Illumina MiSeq to authenticate eighteen botanical preparations labeled as containing *Silybum marianum* (L.) Gaertn. (milk thistle) either as a unique ingredient or in combination with other plant-based ingredients. The results confirm that DNA metabarcoding using Illumina MiSeq can be used to test for the presence of *S. marianum* and simultaneously to detect other plant ingredients within complex herbal preparations with results to be interpreted in a broad context. It should be emphasized however that DNA metabarcoding detected milk thistle in only eleven out of sixteen retained preparations, and the other two had incomplete evidence of milk thistle despite metabolomics validating its presence, challenging its use as a stand-alone approach for routine screening. Moreover, the high sensitivity of DNA metabarcoding requires careful consideration of the total species composition detected by interpreting the results in a broad context, particularly concerning the detection of false positives versus possible contaminants and adulterants. Further, DNA metabarcoding does not provide information on the active metabolites of the botanical preparations, and this narrows its analytical capabilities to the identification of target species and confirmation of presence, but not the absence of other species. The clear advantage of semi-targeted metabolomics based on UHPLC-QTOF-ESI^+^MS consisted in the analytical ability to detect the quantity of the predefined set of phytochemical markers compounds and showing clearly that all investigated milk thistle preparations contained molecules from silymarin complexes at different concentrations. Moreover, metabolomics realized a wider coverage of the relative levels of other metabolites enabling the comparison and discrimination between the different groups of formulations without having *a priori* information about their content. This study shows that the combination of complementary methods offers a robust analytical approach to advance authentication and quality control of botanical preparations.

## Electronic supplementary material

Below is the link to the electronic supplementary material.

Supplementary Material 1

Supplementary Material 2

Supplementary Material 3

Supplementary Material 4

Supplementary Material 5

Supplementary Material 6

Supplementary Material 7

Supplementary Material 8

## Data Availability

The DNA sequencing read data generated and analyzed during the current study are available in Zenodo 10.5281/zenodo.7948441.

## List of Abbreviations

MT: Milk thistle
UHPLC-QTOF-ESI^+^MS: ultra-high-performance liquid chromatography coupled with electrospray -quadrupole-time of flight-mass spectrometry using the positive ionization
UV-VIS: Ulraviolt visible
d.m.: Dry matter
QC: Quality control
m/z: Mass-to-charge ratio
PCA: Principal Component Analysis
sPLSDA: Sparse Partial Least Square Discriminant Analysis
RF: Random Forest
MDA: Mean Decrease Accuracy
PCR: Polymerase chain reaction
nrITS: Nuclear ribosomal internal transcribed spacer
ZOTU: Zero-radius operational taxonomic unit
