# Supplementary material for: Authentication of milk thistle commercial products using UHPLC-QTOF-ESI + MS metabolomics and DNA metabarcoding
Source: BMC Complement Med Ther. 2023 Jul 21;23:257. doi: 10.1186/s12906-023-04091-9 (PMC10360273; doi:10.1186/s12906-023-04091-9)
Supplement: Supplementary file 2 — Supplementary Material 2 [file 12906_2023_4091_MOESM2_ESM.docx]

**Additional Figure 2.A.** Calibration of silybin A+B based on UV spectrometry (max. 287.3 nm)

**Additional Figure 2.B.** Calibration of silybin A+ B based on UHPLC-MS measurements
