# Supplementary material for: Authentication of milk thistle commercial products using UHPLC-QTOF-ESI + MS metabolomics and DNA metabarcoding
Source: BMC Complement Med Ther. 2023 Jul 21;23:257. doi: 10.1186/s12906-023-04091-9 (PMC10360273; doi:10.1186/s12906-023-04091-9)
Supplement: Supplementary file 3 — Supplementary Material 3 [file 12906_2023_4091_MOESM3_ESM.docx]

**Additional file 3.** Identification of 63 putative metabolites found in the genuine milk thistle, and different herbal formulations by semitargeted metabolomics (confirmed by Human Metabolomic Database (HMDB ID), see Materials and methods)

| **Category** | ***m/z***  **[M+1]** | **TR**  **(min)** | **Putative Metabolite identification** | **HMDB ID** |
| --- | --- | --- | --- | --- |
| Silymarin complex | 467.1519 | 5.5 | Silyhermin | HMDB0033324 |
|  | 481.1337 | 9.7-9.9 | Dehydrosilybin | HMDB0040513 |
|  | 483.1475 | 8.2-8.3 | Silybin A / B (Silybinin) | HMDB0030583 |
|  | 483.1830 | 9.5 | Silychristin | HMDB0033953 |
|  | 485.1637 | 5.3 | Silydianin | HMDB0030584 |
| Phenolic acids | 171.1025 | 3.5 | Gallic acid | HMDB0005807 |
|  | 165.0093 | 7.3 | Coumaric acid | HMDB0041592 |
|  | 195.1258 | 7.3 | Ferulic acid | HMDB0000954 |
| Terpenoids | 181.1254 | 3.7 | Coniferyl alcohol | HMDB0012915 |
|  | 333.2734 | 9.9-10.1 | Carnosic acid | HMDB0002358 |
|  | 363.3224 | 12.1-12.2 | Secoisolariciresinol | HMDB0013692 |
| Flavonoids | 271.1941 | 12.6 | Apigenin | HMDB0002124 |
|  | 273.1913 | 12.8-13 | Naringenin | HMDB0002670 |
|  | 287.0617 | 5.3 - 5.5 | Luteolin | HMDB0005800 |
|  | 287.1696 | 9.9 - 10.1 | Kaempherol | HMDB0005801 |
|  | 301.2017 | 5.7 - 5.8 | Luteolin 7-methyl ether | HMDB0037339 |
|  | 303.0647 | 4.5 -4.6 | Quercetin | HMDB0005794 |
|  | 305.2182 | 5.1 | Taxifolin | HMDB0303943 |
|  | 307.1361 | 7.3 | Epigallocatechin | HMDB0038361 |
|  | 319.2933 | 12.1-12.4 | Myricetin | HMDB0002755 |
|  | 401.2783 | 11.8 | Campesterol | HMDB0002869 |
|  | 415.3059 | 11.9 | beta-Sitosterol | HMDB0000852 |
|  | 443.3492 | 12.2-12.4 | (Epi)catechin gallate | HMDB0029255 |
|  | 447.3109 | 7.9-8.1 | Apigenin 7-glucuronide | HMDB0240480 |
|  | 449.1248 | 4.4-4.6 | Luteolin 5-glucoside | HMDB0302805 |
|  | 453.1409 | 8.2-8.3 | Catechin 5-glucoside | HMDB0037948 |
|  | 459.3715 | 5.3-5.5 | Epigallocatechin gallate | HMDB0003153 |
|  | 563.3983 | 6.5 | Campesterol glucoside | HMDB0303802 |
|  | 595.2104 | 3.3-3.5 | Cyanidin 3-rutinoside | HMDB0031458 |
|  | 611.3929 | 3.4 | Rutin | HMDB0003249 |
| Fatty acids & derivatives | 229.1471 | 11.4 | Myristic acid C12:0 | HMDB0000806 |
|  | 255.2366 | 10.1 | Palmitoleic acid C16:1 | HMDB0003229 |
|  | 257.2551 | 3.3 | Palmitic acid C16:0 | HMDB0000220 |
|  | 279.2394 | 12.5 - 12.8 | Linolenic acid C18:3 | HMDB0001388 |
|  | 281.2548 | 12.3 - 12.6 | Linoleic acid C18:2 | HMDB0000673 |
|  | 283.2283 | 12.7 12.9 | Oleic acid C18:1 | HMDB0000207 |
|  | 285.1747 | 11 - 11.2 | Stearic acid C18:0 | HMDB0000827 |
|  | 307.2564 | 11.1 - 11.3 | Eicosatrienoic acid C20:3 | HMDB0002925 |
|  | 309.2198 | 10.2 | Eicosadienoic acid C20:2 | HMDB0005060 |
|  | 311.2297 | 9.6 - 9.7 | Eicosenoic acid C20:1 | HMDB0002231 |
|  | 313.2460 | 12.4 - 12.6 | Arachidic acid C20:0 | HMDB0002212 |
|  | 385.1838 | 5.7-5.9 | Mycolipanolic acid (C24:0) | LMFA01020332 |
|  | 297.2506 | 12 | 9-Hydroxylinoleic acid | HMDB0004670 |
|  | 475.3439 | 10.4-10.8 | Myristyl linolenate | LMFA07010107 |
| Acylated aminoacids | 314.3512 | 11.6 - 11.7 | Palmitoylglycine | HMDB0013034 |
|  | 344.3268 | 10.9-11.1 | N-Palmitoyl Serine | HMDB0241932 |
|  | 354.2758 | 8 | N-oleoyl alanine | HMDB0241953 |
| Polar lipids | 342.3041 | 6.0 - 6.1 | Palmitoylcholine | HMDB0240592 |
|  | 343.3058 | 9.5-9.6 | MG(P-18:0/0:0/0:0) | HMDB0011153 |
|  | 353.2552 | 11.1-11.2 | MG(18:3/0:0/0:0) | HMDB0011569 |
|  | 357.1740 | 6.2 | MG(18:1/0:0/0:0) | HMDB0011567 |
|  | 359.3277 | 12.1-12.2 | MG(18:0/0:0/0:0) | HMDB0011131 |
|  | 371.3391 | 10.9-10 | MG(0:0/18:1-O/0:0) | HMDB0260544 |
|  | 380.3501 | 11.8-12.1 | Sphingosine 1-phosphate | HMDB0000277 |
|  | 424.3992 | 12.4-12.5 | LysoPE(14:1/0:0) | HMDB0011501 |
|  | 433.2780 | 9.2-9.4 | LysoPA(18:3/0:0/) | HMDB0114744 |
|  | 435.2718 | 8.5-8.6 | LysoPA(18:2/0:0/) | HMDB0007856 |
|  | 438.3956 | 12-12.6 | LysoPE(P-16:0/0:0) | HMDB0011152 |
|  | 478.2490 | 9.4 | LysoPE(18:2/0:0) | HMDB0011507 |
|  | 482.4256 | 13.2-13.6 | Cer(d18:1/12:0) | HMDB0004947 |
|  | 520.3619 | 12.2 | LysoPC(18:2/0:0) | HMDB0010386 |
|  | 546.4373 | 13.1 | LysoPC(20:3/0:0) | HMDB0010393 |
|  | 604.4326 | 13.2 | PC(20:3-OH/2:0) | HMDB0288901 |
