# Supplementary material for: Authentication of milk thistle commercial products using UHPLC-QTOF-ESI + MS metabolomics and DNA metabarcoding
Source: BMC Complement Med Ther. 2023 Jul 21;23:257. doi: 10.1186/s12906-023-04091-9 (PMC10360273; doi:10.1186/s12906-023-04091-9)
Supplement: Supplementary file 5 — Supplementary Material 5 [file 12906_2023_4091_MOESM5_ESM.docx]

**Additional file 5.** Mean values of MS Peak intensities for herbal products ( teas, tablets, and capsules) with unique ingredients (U) compared to groups with multiple ingredients (M)

| Molecules | MS Peak intensity/104 | | Ratio U/M |
| --- | --- | --- | --- |
|  | Group U | Group M |  |
| Silybin A+B | 1117.496 | 771.598 | 1.448 |
| Silychristin + Silydianin | 612.541 | 373.902 | 1.638 |
| Taxifolin | 234.607 | 96.611 | 2.428 |
| Silyhermin | 5.608 | 1.806 | 3.105 |
| Total | 962.284 | 472.319 | 2.037 |
