# Supplementary material for: Authentication of milk thistle commercial products using UHPLC-QTOF-ESI + MS metabolomics and DNA metabarcoding
Source: BMC Complement Med Ther. 2023 Jul 21;23:257. doi: 10.1186/s12906-023-04091-9 (PMC10360273; doi:10.1186/s12906-023-04091-9)

**Additional file 7.** Presence of *Silybum marianum* L. within the herbal preparations as detected by DNA metabarcoding. **(A)** Preparations grouped by pharmaceutical form; **(B)** Preparations grouped by the number of ingredients - which declared to have MT as a unique ingredient (U) or declared to have multiple ingredients (M).


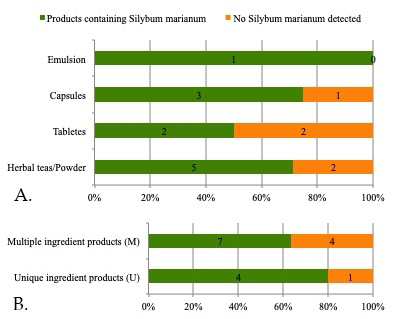

Supplement: Supplementary file 7 — Supplementary Material 7 [file 12906_2023_4091_MOESM7_ESM.docx]
