# Supplementary material for: Authentication of milk thistle commercial products using UHPLC-QTOF-ESI + MS metabolomics and DNA metabarcoding
Source: BMC Complement Med Ther. 2023 Jul 21;23:257. doi: 10.1186/s12906-023-04091-9 (PMC10360273; doi:10.1186/s12906-023-04091-9)

**Additional file 8.** Correspondence between the number of detected (green) and non-detected species (orange) listed on the label of the retained herbal preparations, using DNA metabarcoding.


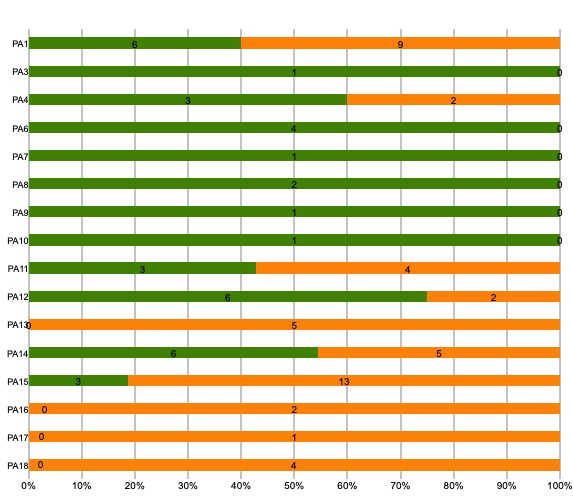

Supplement: Supplementary file 8 — Supplementary Material 8 [file 12906_2023_4091_MOESM8_ESM.docx]
